# Sex differences in hypothalamic astrocyte response to estradiol stimulation

**DOI:** 10.1186/2042-6410-1-7

**Published:** 2010-11-22

**Authors:** John Kuo, Naheed Hamid, Galyna Bondar, Phoebe Dewing, Jenny Clarkson, Paul Micevych

**Affiliations:** 1 Department of Neurobiology, Laboratory of Neuroendocrinology of the Brain Research Institute, David Geffen School of Medicine at UCLA, Los Angeles, CA 90095, USA; 2 Department of Obstetrics and Gynecology, David Geffen School of Medicine at UCLA, Los Angeles, CA 90095, USA

## Abstract

**Background:**

Reproductive functions controlled by the hypothalamus are highly sexually differentiated. One of the most dramatic differences involves estrogen positive feedback, which leads to ovulation. A crucial feature of this positive feedback is the ability of estradiol to facilitate progesterone synthesis in female hypothalamic astrocytes. Conversely, estradiol fails to elevate hypothalamic progesterone levels in male rodents, which lack the estrogen positive feedback-induced luteinizing hormone (LH) surge. To determine whether hypothalamic astrocytes are sexually differentiated, we examined the cellular responses of female and male astrocytes to estradiol stimulation.

**Methods:**

Primary adult hypothalamic astrocyte cultures were established from wild type rats and mice, estrogen receptor-α knockout (ERKO) mice, and four core genotype (FCG) mice, with the sex determining region of the Y chromosome (*Sry*) deleted and inserted into an autosome. Astrocytes were analyzed for *Sry *expression with reverse transcription PCR. Responses to estradiol stimulation were tested by measuring free cytoplasmic calcium concentration ([Ca^2+^]_i_) with fluo-4 AM, and progesterone synthesis with column chromatography and radioimmunoassay. Membrane estrogen receptor-α (mERα) levels were examined using surface biotinylation and western blotting.

**Results:**

Estradiol stimulated both [Ca^2+^]_i _release and progesterone synthesis in hypothalamic astrocytes from adult female mice. Male astrocytes had a significantly elevated [Ca^2+^]_i _response but it was significantly lower than in females, and progesterone synthesis was not enhanced. Surface biotinylation demonstrated mERα in both female and male astrocytes, but only in female astrocytes did estradiol treatment increase insertion of the receptor into the membrane, a necessary step for maximal [Ca^2+^]_i _release. Regardless of the chromosomal sex, estradiol facilitated progesterone synthesis in astrocytes from mice with ovaries (XX and XY^-^), but not in mice with testes (XY^-^*Sry *and XX*Sry*).

**Conclusions:**

Astrocytes are sexually differentiated, and in adulthood reflect the actions of sex steroids during development. The response of hypothalamic astrocytes to estradiol stimulation was determined by the presence or absence of ovaries, regardless of chromosomal sex. The trafficking of mERα in female, but not male, astrocytes further suggests that cell signaling mechanisms are sexually differentiated.

## Background

Sex differences affect the physiological function of both gonadal and non-gonadal cellular systems. When gene expression was studied by microarray in a large number of mice, 55 to 72% of active genes showed sexual dimorphism in the liver, fat and muscle, and 13% of genes were sexually dimorphic in the brain [1]. These sex differences influence a variety of neural functions, both physiological and pathological.

One of the most robust sex differences is the estrogen-positive feedback, which signals the luteinizing hormone (LH) surge essential for ovulation. In post-pubertal females, rising levels of estradiol originating from developing ovarian follicles peak on proestrus, and induce the gonadotropin releasing hormone (GnRH) regulatory circuit to massively release GnRH, which stimulates estrogen primed gonadotrophs to release LH, resulting in ovulation and the formation of corpora lutea. Males, especially male rodents, do not exhibit this phenomenon. Their relatively constant levels of testosterone produce a negative feedback on the regulatory circuitry for GnRH release from the hypothalamus and gonadotropin release from the pituitary, an effect similar to that in females outside of proestrus. The inability of males to produce the estrogen positive feedback leading to a surge in LH has been attributed to the effects of androgen action on the central nervous system [2-7].

A mechanism for mediating estrogen positive feedback involves the synthesis of neuroprogesterone in the hypothalamus. Estradiol treatment of ovariectomized and adrenalectomized female rats increased hypothalamic progesterone levels and induced an LH surge [8]. Disruption of central (hypothalamic) progesterone synthesis blocked the LH surge in gonadally intact, cycling rats [9]. Interestingly, only adult females, which have an estrogen positive feedback mechanism, show an increase in hypothalamic progesterone in response to estradiol [8,10]. In other words, males and reproductive senescent females do not show an increase in hypothalamic progesterone synthesis. The cells responsible for the elevated neuroprogesterone levels in the hypothalamus after estradiol treatment are astrocytes [11].

In astrocytes from post-pubertal female rats, estradiol induces a rapid increase in free cytoplasmic calcium concentration ([Ca^2+^]_i_) that facilitates progesterone synthesis essential for positive estrogen feedback, the LH surge and ovulation in females [10,12-16]. We have not determined whether astrocytes derived from male rats similarly respond to estradiol stimulation by increasing [Ca^2+^]_i _release and progesterone synthesis. The present experiments were performed to determine whether astrocytes derived from male and females respond differently to estradiol stimulation. In addition, *Sry *is expressed in the brain, and has been shown to directly influence the biochemical properties of the dopaminergic neurons of the nigrostriatal system and the specific motor behaviors they control [17]. To this end, the 'four core genotype' (FCG) mouse model, in which the sex chromosome complement is independent of gonadal phenotype [18], was used to determine whether sex differences are due to direct sex chromosome effects or to *Sry *transgene effects that determine gonadal differentiation and its dramatic influence on the sex steroid environment during early development.

## Methods

All experimental procedures were approved by the Chancellor's Animal Research Committee at the University of California at Los Angeles.

### Primary cell cultures

Primary hypothalamic astrocyte cultures pooled from two to six animals were obtained from 50-day-old adult Long-Evans rats (Charles River, Wilmington, MA, USA) and from 60-day-old adult mice (C57/Bl6 wild type and estrogen receptor-alpha (ERα) knockout (Jackson Laboratory, Bar Harbor, ME, USA) and C57BL/6J FCG mice (gift from Dr. Arthur Arnold, UCLA, Los Angeles, CA, USA)). FCG mice were obtained by breeding XX female mice with XY^-^*Sry *male mice, which possess a Y chromosome with the *Sry *gene deleted and a functional *Sry *transgene inserted onto an autosome. The presence of the *Sry *gene leads to differentiation of the indeterminate gonads into testes, and its absence results in formation of ovaries [19,20]. This cross generates four genotypes: XY^-^*Sry *gonadal males (XYM), XY^- ^gonadal females (XYF), XX*Sry *gonadal males (XXM) and XX gonadal females (XXF) [21].

The hypothalamus was dissected with the following boundaries: rostral extent of the optic chiasm, rostral extent of the mammillary bodies, lateral edges of the tuber cinereum and the top of the third ventricle. Hypothalamic tissue was dissociated with 2.5% trypsin solution (Invitrogen, Eugene, OR, USA) and a fire polished glass Pasteur pipette. Cultures were grown in Dulbecco's modified Eagle's medium (DMEM)/F12 (Mediatech, Manassas, VA, USA) with 10% fetal bovine serum (FBS) (Hyclone, Logan, UT, USA) and 1% penicillin (10,000 IU/ml)-streptomycin (10,000 μg/ml) solution (PS) (Mediatech) at 37°C in 5% CO_2_. Astrocyte cultures were grown to confluency and purified from other glial cells [14-16] using a technique modified from McCarthy and de Vellis [22]. *Sry *expression in the astrocytes of FCG mice was analyzed and confirmed by reverse transcription (RT)-PCR.

Before the experiments, the DMEM/F12 medium with 10% FBS and 1% PS was removed, and primary astrocyte cultures were washed with Hanks' balanced salt solution (HBSS) (Mediatech), dissociated with a 2.5% trypsin solution and resuspended in DMEM/F12 medium with 10% FBS. Astrocytes were centrifuged for 3 minutes at 80 *g*, then the supernatant was removed and the pellet containing astrocytes resuspended. Astrocytes were counted, plated and incubated in DMEM/F12 medium with 10% FBS and 1% PS at 37°C in 5% CO_2 _for 24 to 48 hours before Ca^2+ ^imaging and progesterone radioimmunoassay (RIA). For biotinylation, astrocytes were counted, plated and grown in flasks for 2 weeks before experimentation. Cultures were routinely checked for purity using immunocytochemistry for glial fibrillary acidic protein (Chemicon, Temecula, CA, USA) with Hoechst 3342 nuclear stain (Sigma-Aldrich, St. Louis, MO, USA). Cultures were determined to be > 95% pure astrocytes, as previously reported [14-16],

### Intracellular Ca^2+ ^measurements

Astrocytes (5000) were plated onto 15 mm glass coverslips coated with 0.1 mg/ml poly-D lysine (Sigma-Aldrich) in 12-well culture plates and grown in DMEM/F12 medium with 10% FBS and 1% PS at 37°C in 5% CO_2 _for 24 to 48 hours. The astrocytes were then starved of steroid for 18 hours by incubating in DMEM/F12 medium with 5% charcoal-stripped FBS at 37°C in 5% CO_2_. Before imaging, astrocytes were incubated for 45 minutes with HBSS and 4.5 μmol/l of the calcium indicator Fluo-4 AM (Invitrogen) dissolved in dimethyl sulfoxide (DMSO) and methanol. Glass coverslips were placed into a 50 mm chamber insert (Warner Instruments, Hamden, CT, USA) fixed into a 60 × 15 mm cell culture dish (Corning Inc., Corning, NY, USA) and placed into a quick exchange platform (QE-2; Warner Instruments) for imaging under a laser confocal microscope (Axioplan2-LSM 510 Meta; Zeiss, Thornwood, New York, NY, USA). Astrocytes were gravity perfused with HBSS, and media were removed by vacuum suction. Cyclodextrin encapsulated 17β-estradiol (1 nmol/l) (Sigma-Aldrich) was prepared in HBSS and used to induce [Ca^2+^]_i _release. Controls were stimulated with HBSS only. For Fluo-4 AM imaging, a water immersion objective (IR-Achroplan 40X/0.80; Zeiss, Jena, Germany) was used with 488 nm laser excitation and emission monitored through a low-pass filter with a cutoff at 505 nm. The increase in Ca^2+ ^fluorescence (relative fluorescence units (RFU)) was calculated for each astrocyte as the difference between baseline fluorescence and peak response to drug stimulation.

### Progesterone RIA

Approximately 500,000 astrocytes were plated into six-well culture plates and grown for 24 hours. Astrocytes were then starved of steroid in DMEM/F12 medium with 5% charcoal-stripped FBS for 18 hours before treatment with cyclodextrin encapsulated 17β-estradiol (1 or 100 nmol/l dissolved in HBSS) (Sigma-Aldrich) or HBSS with no steroids for 60 minutes at 37°C. After 1 hour of drug treatment, the supernatant for each well was collected and frozen at -20°C for up to 1 week before the RIA.

For the progesterone assay, samples were thawed, mixed with diethyl ether (Fisher Scientific, Fair Lawn, NJ, USA) and then mixed by vortex for 2 minutes. To freeze the aqueous layer, samples were placed into a methanol and dry ice bath. The upper ether layer was decanted into a separate tube and the ether was allowed to evaporate overnight. The extract was reconstituted in isooctane (Mallinkrodt Baker, Phillipsburg, NJ, USA) and a diatomaceous earth column (Celite column™; Celite Corp., Lompoc, CA, USA) with ethylene glycol as the stationary phase was used to isolate the progesterone. Progesterone was then eluted off the column using 4 ml of isooctane. Standards and samples (100 μl) were incubated with rabbit polyclonal antibody against progesterone (Sigma-Aldrich) for 30 minutes at 37°C. Tritium radiolabeled progesterone (2000 counts/minute/ml) was added and incubated for an additional 60 minutes at 37°C. Standards and samples were cooled to 4°C, and a 0.05% charcoal dextran solution (Sigma-Aldrich) added to remove all unbound progesterone. The mixture was centrifuged at 1500 × *g *for 15 minutes at 4°C. The supernatant was then collected for chromatographic detection of progesterone. All samples were run in duplicate, and sample progesterone concentrations determined by extrapolation from a curve determined from the progesterone standards.

### Surface biotinylation

Primary astrocyte cultures were starved of steroids in DMEM/F12 medium with 5% charcoal-stripped FBS 12 hours before treatment with vehicle or 1 nmol/l of 17β-estradiol (Sigma-Aldrich) for 30 minutes at 37°C. Cells in each flask were washed three times with ice cold phosphate buffered saline (PBS) and incubated with freshly prepared biotin (0.5 mg/ml) (EZ-Link Sulfo-NHS-LC-Biotin; Pierce Biotechnology Inc., Rockford, IL, USA) in PBS for 30 minutes at 4°C with gentle agitation. Excess biotin reagent was quenched by rinsing the cells three times with ice cold glycine buffer (50 mmol/l glycine in PBS). Cells were scraped into 10 ml of PBS solution, transferred into a 50 ml conical tube and centrifuged at 500 × *g *for 3 minutes. The pellet was washed twice with ice-cold PBS and resuspended in 200 ml radioimmunoprecipitation assay (RIPA) lysis buffer containing the protease inhibitors 1 mmol/l phenylmethylsulfonyl fluoride, 1 mmol/l EDTA, 1 μg/ml pepstatin, 1 μg/ml leupeptin, 1 μg/ml aprotinin and 1 mmol/l sodium orthovanadate (all from Santa Cruz Biotechnology, Santa Cruz, CA, USA). The cells were homogenized by passing them through a 25-gauge needle, and the cell extract was centrifuged at 10,000 × *g *for 2 minutes at 4°C. The protein concentration of the supernatant was determined using the Bradford Assay (Bio-Rad, Hercules, CA, USA). Samples with equal protein concentration were added to a washed immobilized gel (NeutrAvidin™Gel; Pierce Biotechnology Inc.) for 2 hours at room temperature and then centrifuged at 1,000 × *g *for 1 minute. The beads were washed four times with 1 ml of RIPA buffer (Santa Cruz Biotechnology) containing the protease inhibitors previously mentioned. The bound proteins were eluted with SDS-PAGE sample buffer supplemented with 50 mmol/l dithiothreitol (DTT) for 1 hour at 37°C.

### Western blotting

Samples were separated in a 10% Tris-HCl gel (Ready Gel; Bio-Rad) and transferred to polyvinylidene fluoride membranes (GE Healthcare, Piscataway, NJ, USA). ERα was detected with primary rabbit polyclonal antibody (1:1000) (C1355; Upstate Biotechnology, Inc., Lake Placid, NY, USA). β-actin was used as a loading control and detected by rabbit polyclonal antibody (1:5000) (Abcam, Cambridge, MA, USA). A secondary donkey anti-rabbit IgG (H+L) antibody (1:5000) (Jackson ImmunoResearch, West Grove, PA, USA) and an anti-biotin horseradish peroxidase-linked antibody (1:1000) (Cell Signaling Technology, Danvers, MA, USA) were then used (1.5 hour incubation). To estimate the molecular weight, samples were run alongside a biotinylated protein ladder (Cell Signaling Technology). Immunoreactive bands were visualized using an enhanced chemoluminesence (ECL) kit and ECL hyperfilm (GE Healthcare). Routine exposures varied from 0.5 to 2 minutes. The optical density of each immunoreactive band was determined. For each sample, immunoreactive ERα was normalized with β-actin to obtain the percentage of ERα protein to β-actin protein ratio (% relative ratio).

### RT-PCR

Total RNA was extracted from several primary cultures of astrocytes from Long-Evans post-pubertal rats using Trizol reagent (Invitrogen), following the manufacturer's recommended protocol. To prevent DNA contamination, RNA was treated with DNase I (Ambion, Austin, TX, USA) at 37°C for 30 minutes followed by inactivation with DNase inactivation reagent (Ambion). Total RNA quality and concentration were assessed using a spectrophotometer (NanoDrop 1000; Thermo Scientific, Wilmington, DE, USA). RT was then performed using 1 μg of total RNA to synthesize single-stranded cDNA in a 20 μl reaction with a reverse transcriptase (SuperScript III; Invitrogen) and a combination of random hexamers and oligo (dT)_20 _primers, following the manufacturer's protocol. Briefly, RT was performed at 50°C for 50 minutes, then the reaction terminated at 85°C for 5 minutes and the RNA destroyed with 1 μl of RNase H at 37°C for 20 minutes. cDNAs were subjected to PCR with primers specific to rat *Sry *(Fwd: 5-GCAGCGTGAAGTTGCCTCAAC-3 and Rev: 5-TGCAGCTCTAGCCCAGTCCTG-3) in an RT-PCR system (Mx3000p Real-Time PCR System; Stratagene, Santa Clara, CA, USA). PCR conditions used for amplification were as follows: initial denaturation at 94°C for 10 minutes, 35 cycles of denaturation at 94°C for 45 seconds, annealing at 60°C for 45 seconds and elongation at 72°C for 1 minute, with a final extension at 72°C for 7 minutes. Amplified products were separated by electrophoresis in a 2% agarose gel with ethidium bromide and visualized with ultraviolet light. Gel images were captured digitally to confirm product size and the absence of non-specific products. Negative controls (no cDNA) were included in every PCR run.

### Statistical analysis

Data are presented as means ± standard error (SEM) in RFU, pg/ml or % relative ratio. Statistical comparisons were made using one-way analysis of variance (ANOVA) with Student-Newman-Keuls *post hoc *test when comparing means across at least three independent groups. For the FCG mice, mean comparisons and contrasts under the ANOVA model were made using the Tukey-Fisher least significant difference (LSD) criterion. Statistical calculations were carried out using SAS (version 9.2; SAS Institute, Cary, NC, USA) and GraphPad Prism (version 5; GraphPad Software, La Jolla, CA, USA) software programs. *P *< 0.05 was considered significant.

## Results

### Sex differences of [Ca^2+^]_i _release in response to estradiol stimulation

**Figure 1:**
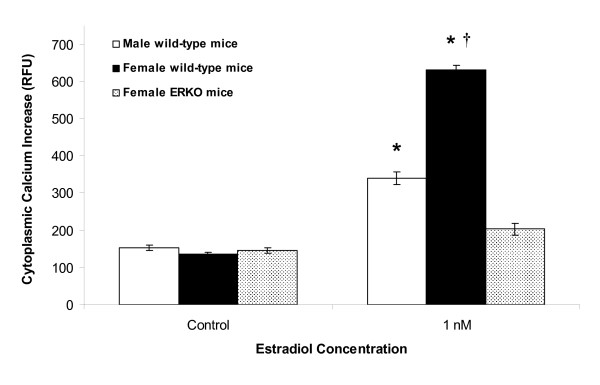
**Gender and estrogen receptor-α affect the estradiol-induced [Ca^2+^]_i _release in adult hypothalamic astrocytes**. The estradiol (1 nmol/l)-induced [Ca^2+^]_i _release was greater in female compared with male wild type astrocytes (*P *< 0.05). In female estrogen receptor-α knockout (ERKO) astrocytes, 1 nmol/l estradiol failed to induce a significant [Ca^2+^]_i _response (*P *> 0.05) [12]. *Significantly different compared with all controls and female ERKO mice (*P *< 0.05, one-way ANOVA with Student-Newman-Keuls *post hoc *test). †Significantly different compared with male wild type astrocytes stimulated with 1 nmol/l estradiol (*P *< 0.05, one-way ANOVA with Student-Newman-Keuls *post hoc *test).

Calcium imaging was used to confirm the sexual differentiation of hypothalamic astrocyte function in adult mice. Although 1 nmol/l estradiol induced a significant (*P *< 0.05) [Ca^2+^]_i _response in both female and male wild type astrocytes (ΔF Ca^2+ ^= 630 ± 13 RFU (n = 31) and ΔF Ca^2+ ^= 340 ± 17 RFU (n = 24), respectively) compared with control (ΔF Ca^2+ ^= 135 ± 6 RFU (n = 21) and ΔF Ca^2+ ^= 152 ± 7 RFU (n = 24), respectively), the estradiol-induced [Ca^2+^]_i _release in male astrocytes was significantly(*P *< 0.05) smaller than the [Ca^2+^]_i _release in female astrocytes (Figure 1). We previously reported that the estradiol-induced [Ca^2+^]_i _response seen in female wild type astrocytes was abolished in female hypothalamic astrocytes from estrogen receptor-α knockout (ERKO) mice [12].

### Sex differences in astrocytic progesterone synthesis to estradiol stimulation

**Figure 2:**
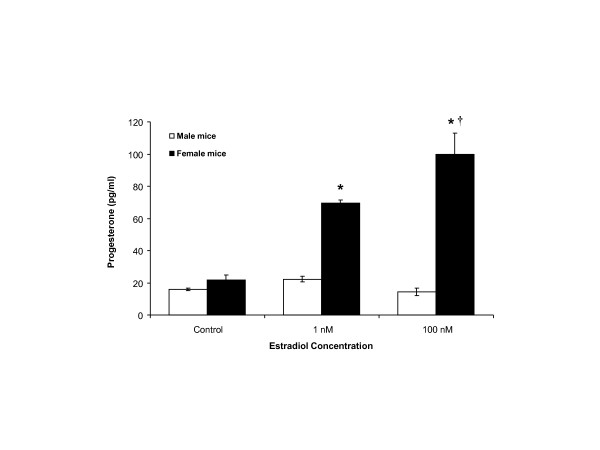
**Sex differences in estradiol stimulated progesterone synthesis in adult hypothalamic astrocytes**. Estradiol at 1 and 100 nmol/l stimulated significant progesterone synthesis in female wild type astrocytes (*P *< 0.05 versus control). Conversely, male wild type astrocytes were not stimulated by estradiol (1 or 100 nmol/l) to synthesize progesterone (*P *> 0.05 versus controls). *Significantly different compared with female control and all male groups (*P *< 0.05, one-way ANOVA with Student-Newman-Keuls *post hoc *test). †Significantly different compared with female astrocytes stimulated with 1 nmol/l estradiol (*P *< 0.05, one-way ANOVA with Student-Newman-Keuls *post hoc *test).

We previously demonstrated that 1 nmol/l estradiol significantly increased progesterone synthesis in primary cultures of adult female hypothalamic astrocytes in rats [12]. Using the same culture conditions in adult female hypothalamic astrocytes obtained from mice, we now confirm that 1 and 100 nmol/l estradiol stimulates significant (*P *< 0.05) progesterone synthesis (69.4 ± 2.0 pg/ml (n = 4) and 99.9 ± 13.0 pg/ml (n = 4), respectively) compared with control (21.6 ± 3.3 pg/ml (n = 4)) (Figure 2). Furthermore, 100 nmol/l estradiol stimulated greater progesterone synthesis compared with 1 nmol/l estradiol (*P *< 0.05) (Figure 2). However, hypothalamic astrocytes from adult male mice did not synthesize progesterone above control levels (15.8 ± 0.8 pg/ml (n = 4)) when stimulated with estradiol at 1 or 100 nmol/l (22.2 ± 1.7 pg/ml (n = 4; *P *> 0.05 versus control) and 14.3 ± 2.4 pg/ml (n = 4; *P *> 0.05 versus control), respectively) (Figure 2). Adult male rat hypothalamic astrocytes similarly failed to synthesize progesterone when exposed to 1 or 100 nmol/l estradiol (20.0 ± 0.6 pg/ml (n = 4) and 18.9 ± 2.4 pg/ml (n = 4), respectively) compared with control (12.7 ± 2.9 pg/ml (n = 4; *P *> 0.05)). These results demonstrate sexual differentiation of hypothalamic astrocyte function in response to estradiol stimulation.

### Sex differences do not result from the sex chromosome complement

**Figure 3:**
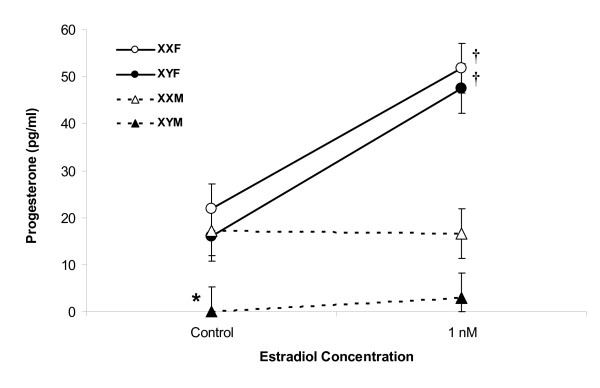
**Sex differences due to gonadal hormone effects in adult hypothalamic astrocytes**. In four core genotype (FCG) mice, control astrocytes from XYM mice synthesized significantly less progesterone (*P *< 0.05) than control astrocytes from XXF, XYF and XXM mice. XXF and XYF astrocytes responded to estradiol (1 nmol/l) with increased progesterone synthesis (*P *< 0.05 versus their control), but XXM and XYM astrocytes did not (*P *> 0.05 versus their control). *Significantly different compared with all other controls (*P *< 0.05, ANOVA with Tukey-Fisher least significant difference *post hoc *test). †Significantly greater progesterone synthesis with 1 nmol/l estradiol compared with the control (*P *< 0.05, ANOVA with Tukey-Fisher least significant difference *post hoc *test).

Using FCG mice, we compared genetic sex chromosome effects versus *Sry *transgene effects. Baseline progesterone synthesis by control astrocytes from XYM mice (0.0 ± 5.3 pg/ml (n = 4)) was significantly lower than control astrocytes from XXF (21.8 ± 5.3 pg/ml (n = 4; *P *< 0.05)), XYF (16.0 ± 5.3 pg/ml (n = 4; *P *< 0.05)) and XXM mice (17.2 ± 5.3 pg/ml (n = 4; *P *< 0.05)), all of which were similar to each other (*P *> 0.05) (Figure 3). Estradiol (1 nmol/l) significantly increased progesterone synthesis in both types of gonadal female (XXF and XYF) astrocytes (51.9 ± 5.3 pg/ml (n = 4; *P *< 0.05 versus control) and 47.4 ± 5.3 pg/ml (n = 4; *P *< 0.05 versus control), respectively), but failed to increased progesterone synthesis in gonadal male (XXM and XYM) astrocytes (16.6 ± 5.3 pg/ml (n = 4; *P *> 0.05 versus control) and 3.0 ± 5.3 pg/ml (n = 4; *P *> 0.05 versus control), respectively) (Figure 3). Comparison of progesterone changes after estradiol (1 nmol/l) versus control revealed a similar increase in progesterone synthesis in XXF and XYF astrocytes, whereas there was a lack of progesterone increase in both XXM and XYM astrocytes (*P *< 0.05), suggesting a steroid-induced sex effect due to early differential gonadal (testes versus ovaries) hormone secretion.

### Sex differences in astrocytic mERα trafficking in response to estradiol

**Figure 4:**
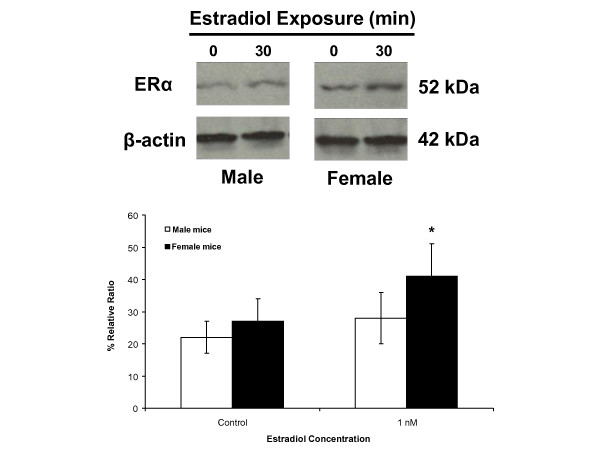
**Sex differences in the regulation of mERα levels in adult hypothalamic astrocytes**. Using surface biotinylation, hypothalamic astrocytes from female and male wild type mice have similar levels of baseline estrogen receptor-α (ERα) at the cell membrane (*P *> 0.05). Upon exposure to 1 nmol/l estradiol for 30 minutes, mERα levels significantly increased in the female astrocytes (*P *< 0.05 versus controls). However, estradiol did not regulate mERα concentration in the male astrocytes (*P *> 0.05 versus controls). *Significantly different compared with all other groups (*P *< 0.05, one-way ANOVA with Student-Newman-Keuls *post hoc *test).

We previously used surface biotinylation to demonstrate the presence of two ERα immunoreactive bands (66 kDa and 52 kDa) in the cell membrane of female wild type hypothalamic astrocytes, which were not present in female ERKO mouse astrocytes [23]. Immunoreactive mERα is transiently increased by estradiol exposure, reaching maximum levels after 30 minutes [23]. As in the previous study, the major ERα immunoreactive band was at 52 kDa, and was thus used for quantification. Basal levels of mERα were similar in both female and male wild type hypothalamic mouse astrocytes (27 ± 7% relative ratio (n = 3) and 22 ± 5% relative ratio (n = 3), respectively; *P *> 0.05) (Figure 4). Stimulation with 1 nmol/l estradiol for 30 minutes significantly increased mERα levels in female astrocytes (41 ± 10% relative ratio (n = 3; *P *< 0.05 versus control)). An equimolar concentration of estradiol did not change mERα levels in male astrocytes (28 ± 8% relative ratio (n = 3; *P *> 0.05 versus control)) (Figure 4). These results demonstrate a sex difference in the regulation of mERα trafficking in response to estradiol exposure in hypothalamic astrocytes.

## Discussion

Although both male and female rodents have a well developed negative feedback mechanism regulating the release of GnRH and LH, one of the most robust sexually differentiated physiological responses is estrogen positive feedback, which induces the surge release of LH in response to estradiol stimulation. This phenomenon of estrogen positive feedback is a hallmark of various female animal species. For rodents, once the ability to produce estrogen positive feedback is lost during development, the deficit is permanent. In primates, including humans, many years of continuous estrogen exposure in males can result in an estrogen positive feedback response, although it is much attenuated [24]. According to the epigenetic theory of sexual differentiation of the brain, the sex difference in estrogen positive feedback is said to arise from the action of estradiol (aromatized from testosterone) during organization of the neural circuit(s) controlling GnRH neurons. Several mechanisms have been proposed to account for this differentiation, including the lack of estrogen-induced synaptic plasticity in the male arcuate nucleus [25] and an attenuated distribution of kisspeptin neurons in males [26]. Various structural sex differences that result from perinatal exposure to estradiol have been identified. In terms of regulating GnRH, males have greater postnatal apoptosis in the developing anteroventral periventricular nucleus (AVPV), a region crucial for estrogen positive feedback in females [27-29]. Although it is not clear whether such a sex difference in apoptosis is an important mechanism for elimination of estrogen positive feedback, it does support a role for postnatal sex steroids in organizing brain mechanisms involved in reproduction [30].

Over the past several years we have been investigating the role of neuroprogesterone in regulating estrogen positive feedback [31]. Experimental evidence is consistent with the hypothesis that synthesis of progesterone in the hypothalamus is needed for an LH surge and the transition from proestrus to estrus [8,10]. Significantly, estrogen treatment increases progesterone levels in the female but not male hypothalamus [8,31]. The cells responsible for neuroprogesterone production are hypothalamic astrocytes [11]. It has been reported that astrocytes are sexually differentiated by the neonatal hormonal milieu, especially estradiol, which profoundly influences their morphology and function [32,33]. Female astrocytes express ERs that are targeted to the cell membrane and directly regulated by estradiol levels [13,23]. Estradiol stimulated mERα transactivates the metabotropic glutamate receptor (mGluR) type 1a, which activates a phospholipase C-inositol trisphosphate (PLC-IP_3_) cascade, resulting in the release of intracellular Ca^2+ ^from the smooth endoplasmic reticulum via an IP_3 _receptor dependent mechanism [13,15]. The robust increase of [Ca^2+^]_i _release stimulates neuroprogesterone synthesis in hypothalamic astrocytes, which is required for the LH surge [11]. Alternatively, astrocytes can potentially modulate GnRH neurons in the arcuate nucleus directly through ensheathment or unensheathment of synaptic connections or restriction of GnRH nerve terminal access to the portal vasculature, and indirectly through release of growth factors and prostagladins [34,35].

In the present study, we investigated astrocytic sex differences by examining the response to estradiol of adult hypothalamic astrocytes derived from male or female rats and mice. First, both male and female astrocytes have mERα that can be labeled using surface biotinylation [23]. Estradiol exposure increased the amount of ERα at the cell membrane in adult female astrocytes, but did not increase levels of mERα in male astrocytes. Second, male astrocytes did have an estradiol-induced [Ca^2+^]_i _response, which was significantly attenuated compared with that of female astrocytes. These results are consistent with our previous observations that maximum [Ca^2+^]_i _release is correlated with elevations in estradiol-induced mERα levels [23]. The importance of mERα was emphasized by the lack of estradiol-induced [Ca^2+^]_i _release in female ERKO astrocytes [12]. This stimulation of [Ca^2+^]_i _release involved mERα transactivation of mGluR1a, a G protein coupled receptor [15]. Furthermore, the estradiol-induced progesterone synthesis in adult female astrocytes similarly required this mERα-mGluR1a interaction [12]. Third, only astrocytes derived from adult female rats [10,12,14] and mice (present results) had an increase in progesterone synthesis, consistent with previous observations that only adult female rodents have increased levels of progesterone in the hypothalamus before the LH surge [8]. These results support the hypothesis that estrogen positive feedback requires a robust [Ca^2+^]_i _release that triggers progesterone synthesis in the hypothalamus. The data also suggest that although a rise in [Ca^2+^]_i _is necessary for progesterone synthesis, there appears to be a crucial concentration required, as male astrocytes have an attenuated [Ca^2+^]_i _response that was unable to facilitate progesterone synthesis in the present study, consistent with previous reports in neonatal astrocytes [14] and in post-pubertal astrocytes, in which 0.1 nmol/l estradiol stimulated [Ca^2+^]_i _release, but not progesterone synthesis [12].

Biological differences between males and females can result genetically from direct sex chromosome differences, developmentally through differential exposure to sex steroids during developmental 'organization', or functionally from acute 'activational' effects of gonadal steroids operating at many life stages, which can be controlled through gonadectomy [36]. Perinatal gonadal hormone secretions have been shown to have powerful and permanent actions on physiology, including pituitary function, gene expression in the brain and sexual behavior [37-40]. In spite of these epigenetic effects, several chromosomal dependent sex differences have been demonstrated in the brain. Specifically, the FCG mice model has demonstrated purely chromosomal XX versus XY differences in behaviors, including aggression, parenting, habit formation, nociception and social interactions [21]. For example, a chromosomal sex effect was demonstrated for vasopressin innervation of the septum [41,42]. At embryonic day 13, mesencephalic neurons express tyrosine hydroxylase, which is earlier than any sex steroid actions [43]. More importantly, neurons in the adult male substantia nigra were shown to express *Sry*, which maintained the expression of tyrosine hydroxylase, the rate limiting enzyme of catecholamine (dopamine) synthesis [17]. A reduction in *Sry *gene expression led to motor deficits in male rats, suggesting a function for *Sry *in the maintenance of dopamine neurons needed for motoric behaviors regulated by the nigrostriatal pathway that is affected in Parkinson's disease. These studies suggest that *Sry *directly affects the biochemical properties of the dopaminergic neurons of the nigrostriatal system and the specific motor behaviors they control.

Both male and female astrocytes have mERα, respond to estradiol stimulation by elevating [Ca^2+^]_i _levels and synthesize progesterone. However, only in female astrocytes can estradiol increase the synthesis of progesterone (4- to 6.5-fold), a critical step in estrogen positive feedback [10]. Although we demonstrated a stark difference between male and female astrocytic response to estradiol, it was not clear whether this cellular differentiation was due to differences in the sex chromosome complement or to the presence of the *Sry *transgene with its influence on gonadal development and early sex steroid environment. Astrocytes from FCG mice were used to specifically differentiate the effects of sex chromosomes versus those of the *Sry *transgene. Animals with ovaries (XXF and XYF) had astrocytes in which estradiol facilitated progesterone synthesis, regardless of whether they had one or two X chromosomes. Conversely, mice with testes (XYM and XXM) were unresponsive to estradiol and did not increase progesterone synthesis. These results suggest a *Sry *transgene effect and not a sex chromosome effect on hypothalamic astrocyte response to estradiol. The effects from the *Sry *transgene could be due to direct effects of the *Sry *gene itself or its influence on gonadal differentiation and the sex steroid environment during early development. Interestingly, XYM from FCG mice synthesized little or no progesterone. This may reveal a potential chromosomal effect. However, male wild type astrocytes, without *Sry *translocation to an autosome, synthesized basal progesterone levels similar to female wild type astrocytes. Therefore, this difference could potentially be caused by the deletion and transgenic insertion of *Sry*, resulting in the inactivation of surrounding gene(s), positional effects or differential expression of the *Sry *transgene. Differences between wild type XY males and FCG XYM have been previously reported for mounting behavior, social exploration and concentration of tyrosine hydroxylase-immunoreactive neurons within the AVPV [41]. Unfortunately, the steroid profile of XYM in FCG mice has not yet been characterized and will require further experimentation.

## Conclusions

Although there may have been a hint of a chromosomal sex difference in the basal level of progesterone synthesis, the overwhelming effect appeared to be from the *Sry *transgene, probably from its dramatic influence on gonadal differentiation and the steroid environment during early development. Wild type male astrocytes expressed *Sry*, and were sexually differentiated from wild type female astrocytes in terms of their [Ca^2+^]_i _and progesterone responses to estradiol. These results are consistent with the stark sexual differentiation of estrogen positive feedback, which is dependent on the postnatal gonadal steroid environment. A potential mechanism for this sex difference in estradiol response was the trafficking of mERα in females but not in males. The estradiol-induced transient increase in mERα levels has been correlated with the robust [Ca^2+^]_i _release necessary for progesterone synthesis. These results suggest that cell signaling in hypothalamic astrocytes is sexually differentiated, mainly as a result of postnatal gonadal steroid exposure, which may also mask the influence of possibly minor chromosomal effects.

## Competing interests

The authors declare that they have no competing interests.

## Authors' contributions

JK carried out the intracellular Ca^2+ ^experiments, participated in the design and coordination of the study, performed the statistical analysis and drafted the manuscript. NH performed the primary cell culturing and carried out the progesterone radioimmunoassay experiments. GB performed the primary cell culturing and carried out the surface biotinylation and western blotting experiments. PD carried out the reverse transcription polymerase chain reaction. JC performed the primary cell culturing and participated in the design of the study. PM conceived of the study, participated in its design and coordination and drafted the manuscript. All authors read and approved the final manuscript.

## References

[B1] Yang X Schadt EE Wang S Wang H Arnold AP Ingram-Drake L Drake TA Lusis AJ Tissue-specific expression and regulation of sexually dimorphic genes in mice Genome Res 2006 16 995 1004 10.1101/gr.5217506 16825664 PMC1524872

[B2] Neill JD Sexual differences in the hypothalamic regulation of prolactin secretion Endocrinology 1972 90 1154 1159 10.1210/endo-90-5-1154 5062474

[B3] Rudd CD Short RV McFarlane JR Renfree MB Sexual differentiation of oestradiol LH positive feedback in a marsupial J Reprod Fertil 1999 115 269 274 10.1530/jrf.0.1150269 10434932

[B4] Gorski RA Motta M Sexual differentiation of the endocrine brain and its control Brain Endocrinology 1991 2 New York: Raven Press 71 104

[B5] Gorski RA Sexual dimorphisms of the brain J Anim Sci 1985 61 Suppl 3 38 61 3908433 10.1093/ansci/61.supplement_3.38

[B6] Booth JE Finn CA Sexual differentiation of the brain Oxford Reviews of Reproductive Biology 1979 1 Oxford: Clarendon Press 58 158

[B7] MacLusky NJ Naftolin F Sexual differentiation of the central nervous system Science 1981 211 1294 1302 10.1126/science.6163211 6163211

[B8] Micevych P Sinchak K Mills RH Tao L LaPolt P Lu JK The luteinizing hormone surge is preceded by an estrogen-induced increase of hypothalamic progesterone in ovariectomized and adrenalectomized rats Neuroendocrinology 2003 78 29 35 10.1159/000071703 12869797

[B9] Micevych P Sinchak K Estradiol regulation of progesterone synthesis in the brain Mol Cell Endocrinol 2008 290 44 50 10.1016/j.mce.2008.04.016 18572304 PMC2603025

[B10] Micevych P Soma KK Sinchak K Neuroprogesterone: key to estrogen positive feedback? Brain Res Rev 2008 57 470 480 10.1016/j.brainresrev.2007.06.009 17850878 PMC2647997

[B11] Micevych P Sinchak K Synthesis and function of hypothalamic neuroprogesterone in reproduction Endocrinology 2008 149 2739 2742 10.1210/en.2008-0011 18308840 PMC2408819

[B12] Kuo J Hamid N Bondar G Prossnitz ER Micevych P Membrane estrogen receptors stimulate intracellular calcium release and progesterone synthesis in hypothalamic astrocytes J Neurosci 2010 30 12950 12957 10.1523/JNEUROSCI.1158-10.2010 20881113 PMC2957903

[B13] Chaban VV Lakhter AJ Micevych P A membrane estrogen receptor mediates intracellular calcium release in astrocytes Endocrinology 2004 145 3788 3795 10.1210/en.2004-0149 15131017

[B14] Micevych PE Chaban V Ogi J Dewing P Lu JK Sinchak K Estradiol stimulates progesterone synthesis in hypothalamic astrocyte cultures Endocrinology 2007 148 782 789 10.1210/en.2006-0774 17095591

[B15] Kuo J Hariri OR Bondar G Ogi J Micevych P Membrane estrogen receptor-alpha interacts with metabotropic glutamate receptor type 1a to mobilize intracellular calcium in hypothalamic astrocytes Endocrinology 2009 150 1369 1376 10.1210/en.2008-0994 18948402 PMC2654734

[B16] Sinchak K Mills RH Tao L LaPolt P Lu JK Micevych P Estrogen induces de novo progesterone synthesis in astrocytes Dev Neurosci 2003 25 343 348 10.1159/000073511 14614261

[B17] Dewing P Chiang CW Sinchak K Sim H Fernagut PO Kelly S Chesselet MF Micevych PE Albrecht KH Harley VR Vilain E Direct regulation of adult brain function by the male-specific factor SRY Curr Biol 2006 16 415 420 10.1016/j.cub.2006.01.017 16488877

[B18] Arnold AP Burgoyne PS Are XX and XY brain cells intrinsically different? Trends Endocrinol Metab 2004 15 6 11 10.1016/j.tem.2003.11.001 14693420

[B19] Jost A Vigier B Prepin J Perchellet JP Studies on sex differentiation in mammals Recent Prog Horm Res 1973 29 1 41 4584366 10.1016/b978-0-12-571129-6.50004-x

[B20] Sinclair AH Berta P Palmer MS Hawkins JR Griffiths BL Smith MJ Foster JW Frischauf AM Lovell-Badge R Goodfellow PN A gene from the human sex-determining region encodes a protein with homology to a conserved DNA-binding motif Nature 1990 346 240 244 10.1038/346240a0 1695712

[B21] Arnold AP Chen X What does the "four core genotypes" mouse model tell us about sex differences in the brain and other tissues? Front Neuroendocrinol 2009 30 1 9 10.1016/j.yfrne.2008.11.001 19028515 PMC3282561

[B22] McCarthy KD de Vellis J Preparation of separate astroglial and oligodendroglial cell cultures from rat cerebral tissue J Cell Biol 1980 85 890 902 10.1083/jcb.85.3.890 6248568 PMC2111442

[B23] Bondar G Kuo J Hamid N Micevych P Estradiol induced estrogen receptor-alpha trafficking J Neurosci 2009 29 15323 15330 10.1523/JNEUROSCI.2107-09.2009 19955385 PMC2836237

[B24] Goh HH Ratnam SS The LH surge in humans: its mechanism and sex difference Gynecol Endocrinol 1988 2 165 182 10.3109/09513598809023624 3055821

[B25] Horvath TL Garcia-Segura LM Naftolin F Lack of gonadotropin-positive feedback in the male rat is associated with lack of estrogen-induced synaptic plasticity in the arcuate nucleus Neuroendocrinology 1997 65 136 140 10.1159/000127173 9067991

[B26] Clarkson J Herbison AE Postnatal development of kisspeptin neurons in mouse hypothalamus; sexual dimorphism and projections to gonadotropin-releasing hormone neurons Endocrinology 2006 147 5817 5825 10.1210/en.2006-0787 16959837 PMC6098691

[B27] Herbison AE Multimodal influence of estrogen upon gonadotropin-releasing hormone neurons Endocr Rev 1998 19 302 330 10.1210/er.19.3.302 9626556

[B28] Yoshida M Yuri K Kizaki Z Sawada T Kawata M The distributions of apoptotic cells in the medial preoptic areas of male and female neonatal rats Neurosci Res 2000 36 1 7 10.1016/S0168-0102(99)00100-5 10678526

[B29] Tsukahara S Kakeyama M Toyofuku Y Sex differences in the level of Bcl-2 family proteins and caspase-3 activation in the sexually dimorphic nuclei of the preoptic area in postnatal rats J Neurobiol 2006 66 1411 1419 10.1002/neu.20276 17013925

[B30] Tsukahara S Sex differences and the roles of sex steroids in apoptosis of sexually dimorphic nuclei of the preoptic area in postnatal rats J Neuroendocrinol 2009 21 370 376 10.1111/j.1365-2826.2009.01855.x 19226350

[B31] Micevych P Bondar G Kuo J Estrogen actions on neuroendocrine glia Neuroendocrinology 2010 91 211 222 10.1159/000289568 20332598 PMC2889254

[B32] McCarthy MM Amateau SK Mong JA Steroid modulation of astrocytes in the neonatal brain: implications for adult reproductive function Biol Reprod 2002 67 691 698 10.1095/biolreprod.102.003251 12193373

[B33] Mong JA Blutstein T Estradiol modulation of astrocytic form and function: implications for hormonal control of synaptic communication Neuroscience 2006 138 967 975 10.1016/j.neuroscience.2005.10.017 16326016

[B34] Garcia-Segura LM McCarthy MM Minireview: Role of glia in neuroendocrine function Endocrinology 2004 145 1082 1086 10.1210/en.2003-1383 14670989

[B35] Prevot V Glial-neuronal-endothelial interactions are involved in the control of GnRH secretion J Neuroendocrinol 2002 14 247 255 10.1046/j.0007-1331.2001.00772.x 11999726

[B36] Arnold AP van Nas A Lusis AJ Systems biology asks new questions about sex differences Trends Endocrinol Metab 2009 20 471 476 10.1016/j.tem.2009.06.007 19783453 PMC2787703

[B37] Micevych PE Abelson L Fok H Ulibarri C Priest CA Gonadal steroid control of preprocholecystokinin mRNA expression in the limbic-hypothalamic circuit: comparison of adult with neonatal steroid treatments J Neurosci Res 1994 38 386 398 10.1002/jnr.490380404 7932871

[B38] Ward I Differential effect of pre- and postnatal androgen on the sexual behavior of intact and spayed rats Horm Behav 1969 1 25 36 10.1016/0018-506X(69)90003-8

[B39] Gorski RA Martini L, Ganong WF Gonadal hormones and the perinatal development of neuroendocrine function Frontiers in Neuroendocrinology 1971 New York: Oxford University Press 237 290

[B40] Pfeiffer CA Sexual differences of the hyphophyses and their determination by the gonads Am J Anat 1936 58 195 225 10.1002/aja.1000580112

[B41] De Vries GJ Rissman EF Simerly RB Yang LY Scordalakes EM Auger CJ Swain A Lovell-Badge R Burgoyne PS Arnold AP A model system for study of sex chromosome effects on sexually dimorphic neural and behavioral traits J Neurosci 2002 22 9005 9014 12388607 10.1523/JNEUROSCI.22-20-09005.2002 PMC6757680

[B42] Gatewood JD Wills A Shetty S Xu J Arnold AP Burgoyne PS Rissman EF Sex chromosome complement and gonadal sex influence aggressive and parental behaviors in mice J Neurosci 2006 26 2335 2342 10.1523/JNEUROSCI.3743-05.2006 16495461 PMC6674813

[B43] Sibug R Kuppers E Beyer C Maxson SC Pilgrim C Reisert I Genotype-dependent sex differentiation of dopaminergic neurons in primary cultures of embryonic mouse brain Brain Res Dev Brain Res 1996 93 136 142 10.1016/0165-3806(96)00024-7 8804700

